# Interpretable Machine Learning Model Integrating Electrocardiographic and Acute Physiology Metrics for Mortality Prediction in Critical Ill Patients

**DOI:** 10.3390/jcm14207163

**Published:** 2025-10-11

**Authors:** Qiuyu Wang, Bin Wang, Bo Chen, Qing Li, Yutong Zhao, Tianshan Dong, Yifei Wang, Ping Zhang

**Affiliations:** 1 School of Clinical Medicine, Tsinghua University, Haidian District, Beijing 100084, China; qy-wang23@mails.tsinghua.edu.cn (Q.W.); wang2022@mail.tsinghua.edu.cn (B.W.); chenbocd@hotmail.com (B.C.); lqs01985@btch.edu.cn (Q.L.); zhaoyutong1001@163.com (Y.Z.); 2 Department of Cardiology, Beijing Tsinghua Changgung Hospital, Tsinghua Medicine, Tsinghua University, Beijing 102218, China; 3 School of Astronautics, Beihang University, Haidian District, Beijing 100191, China; tsdong@buaa.edu.cn

**Keywords:** electrocardiogram, multimodal clinical data, machine learning, mortality risk prediction, intensive care unit

## Abstract

**Background**: Critically ill patients in the intensive care unit (ICU) are characterized by complex comorbidities and a high risk of short-term mortality. Traditional severity scoring systems rely on physiological and laboratory variables but lack direct integration of electrocardiogram (ECG) data. This study aimed to construct an interpretable machine learning (ML) model combining ECG-derived and clinical variables to predict 28-day mortality in ICU patients. **Methods**: A retrospective cohort analysis was performed with data from the MIMIC-IV v2.2 database. The primary outcome was 28-day mortality. An ECG-based risk score was generated from the first ECG after ICU admission using a deep residual convolutional neural network. Feature selection was guided by XGBoost importance ranking, SHapley Additive exPlanations, and clinical relevance. A three-variable model comprising ECG score, APS-III score, and age (termed the E3A score) was developed and evaluated across four ML algorithms. We evaluated model performance by calculating the AUC of ROC curves, examining calibration, and applying decision curve analysis. **Results**: A total of 18,256 ICU patients were included, with 2412 deaths within 28 days. The ECG score was significantly higher in non-survivors than in survivors (median [IQR]: 24.4 [15.6–33.4] vs. 13.5 [7.2–22.1], *p* < 0.001). Logistic regression demonstrated the best discrimination for the E3A score, achieving an AUC of 0.806 (95% CI: 0.784–0.826) for the test set and 0.804 (95% CI: 0.772–0.835) for the validation set. **Conclusions**: Integrating ECG-derived features with clinical variables improves prognostic accuracy for 28-day mortality prediction in ICU patients, supporting early risk stratification in critical care.

## 1. Introduction

Critically ill patients admitted to intensive care units (ICUs) often experience dysfunction in multiple organ systems, resulting in a markedly elevated risk of short-term mortality [1,2,3]. Accurately predicting short-term mortality in this heterogeneous population remains a major clinical challenge. Timely identification of patients at high risk enables earlier escalation of care—such as expedited senior review, targeted laboratory or arterial blood gas reassessment, closer physiologic monitoring, and timely initiation or intensification of organ support—while recognition of low-risk patients supports conservative management, step-down planning, and bed allocation. Because ICU resources (staffing, monitored beds, ventilators) are finite and interventions carry iatrogenic risk, prognostic tools are most useful when they provide calibrated, threshold-linked risk estimates that can be aligned with concrete actions in triage and de-intensification pathways.

Several widely used severity scoring systems have been developed to assist in prognostic assessment in ICU settings, including the Acute Physiology Score III (APS III), Simplified Acute Physiology Score II (SAPS II), Sequential Organ Failure Assessment (SOFA), Oxford Acute Severity of Illness Score (OASIS), Systemic Inflammatory Response Syndrome (SIRS), Glasgow coma scale (GCS), and the Charlson Comorbidity Index [4,5,6,7,8]. However, although cardiac abnormalities, such as myocardial ischemia and arrhythmias, are known to significantly impact outcomes in critically ill patients [9,10], most conventional scoring systems rely on indirect parameters (e.g., heart rate, blood pressure, vasopressor use) to infer cardiovascular function. A key limitation of these models is the underutilization of electrocardiogram (ECG) data, which offers a direct, noninvasive, and dynamic assessment of cardiac electrophysiology. Despite being readily available in ICU settings, ECG data have historically been overlooked in predictive modeling due to limitations in feature extraction and interpretability.

ECG is one of the most used and readily accessible diagnostic tools for evaluating cardiac status in critically ill patients. Beyond traditional rhythm interpretation, emerging evidence suggests that ECG-derived features may contain prognostic signals relevant to a variety of clinical outcomes, including those associated with heart failure, chronic obstructive pulmonary disease, and liver disease [11,12,13,14,15]. However, few existing mortality prediction models have systematically incorporated ECG data in a quantitative manner. The complex interpretation of ECG features typically requires specialized cardiology expertise, which limits broader clinical utilization.

Recent advances in artificial intelligence, particularly deep learning, have enabled automated extraction of complex patterns from unstructured data such as medical images and physiological waveforms [14,15]. Simultaneously, the evolution of machine learning (ML) methods has facilitated the integration of high-dimensional, multimodal clinical data, supporting improved outcome prediction and more personalized critical care management [15,16,17].

Accordingly, the present study aimed to develop a machine learning–based model for predicting 28-day mortality in ICU patients by incorporating ECG-derived features alongside clinical variables. To enhance transparency and clinical interpretability, we applied SHapley Additive exPlanations (SHAP) as a means to assess the contribution of each predictor. This approach bridges the gap between routinely collected ECG data and ICU risk stratification, offering a practical and interpretable tool for early mortality prediction in critically ill populations.

## 2. Materials and Methods

### 2.1. Study Design and Data Source

We carried out a retrospective cohort analysis based on data from the Medical Information Mart for Intensive Care IV (MIMIC-IV) database (version 2.2). The MIMIC-IV v2.2 contained the de-identified clinical information of 299,712 patients from ICU or emergency department at the Beth Israel Deaconess Medical Center (BIDMC) between 2008 and 2019 [18]. Data extraction was performed using Structured Query Language (SQL) in PostgreSQL by researcher Yifei Wang, who holds certification from the Collaborative Institutional Training Initiative (CITI) program (ID: 12401699).

### 2.2. Study Population and Endpoints

The study included all adult patients admitted to ICU. The inclusion criteria were: (1) First ICU admission with a stay longer than 24 h; (2) Age ≥ 18 years. Patients without any ECG recordings during their ICU stay were excluded. After applying these criteria, a total of 18,256 patients were included in the final cohort (Figure S1). The primary outcome was 28-day all-cause mortality.

### 2.3. Clinical Feature Extraction and ECG Based Risk Score Generation

The following clinical variables were extracted (Table S1): (1) Demographics: age, sex, race, height, weight, etc.; (2) Vital signs at admission: heart rate, atrial systolic blood pressure, atrial diastolic blood pressure, respiratory rate, pulse oxygen saturation, body temperature, etc.; (3) Laboratory tests: white blood cells, hemoglobin, platelets, red blood cell distribution width, hematocrit, blood urea nitrogen, creatinine, lactate dehydrogenase, fibrinogen, anion gap, etc.; (4) Interventions: use of vasopressors (norepinephrine, dopamine, epinephrine, vasopressin), mechanical ventilation, and continuous renal replacement therapy; (5) Comorbidities: acute myocardial infarction, heart failure, sudden cardiac arrest, stroke, chronic kidney disease, acute kidney injury, sepsis, tumor, hypertension, diabetes mellitus, hyperlipidemia, etc.; (6) Severity scores: SOFA, OASIS, APS-III, SAPS-II, SIRS, GCS and CHARLSON.

We anchored all time-varying predictors to the ICU admission time and restricted extraction to the first 24 h of the ICU stay. To ensure standardized, reproducible definitions, physiologic and laboratory “first-day” features, comorbidity burden (Charlson), and severity scores (APS-III, SAPS-II) were derived using the publicly available MIT-LCP MIMIC-IV concept queries (https://github.com/MIT-LCP/mimic-code/tree/main/mimic-iv, accessed on 16 March 2025). By design, these concepts compute severity scores from the worst measurements within the first ICU day and limit feature windows to that period; measurements obtained in the emergency department (including any “boarding” interval prior to ICU bed availability) are therefore not included. When considered as candidate predictors, early therapies (e.g., vasopressors, mechanical ventilation) were summarized within the same first-24-h window.

**Figure 1:**
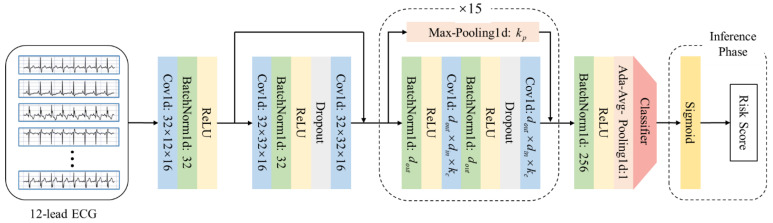
Convolutional neural network architecture based on residual block.

To quantify cardiac risk, we extracted the first ECG recording after ICU admission and developed an ECG-based mortality risk score. A relatively simple neural network model may not be able to effectively extract the key features of ECG. The more complex neural network model may lead to the loss of ECG features in the transmission process, and cause the waste of computing resources. Inspired by Hannun et al. [19], we used a convolutional neural network based on residual block to train and test the ECG (Figure 1). Specifically, the model includes 16 residual blocks, 33 convolutional layers, and 16 pooling layers, and extracts features from the input original ECG. Then the logits value is output by a classifier constructed by a fully connected layer. The dataset for ECG model training was randomly split into training, testing, and validation sets in a 7:2:1 ratio. In the training phase, we use the logits value output by the classifier and the label to calculate the loss value to optimize the model parameters. In the inference phase, we use the sigmoid function to directly process the logits value to obtain the risk score.

### 2.4. Machine Learning Model Development and Validation

We split the cohort into training, test, and validation subsets (7:2:1), identical to the deep-learning pipeline. The ECG-derived score was entered on its original scale without transformation. Tree-based models used XGBoost (XGBClassifier, version 2.1.3) with package defaults, which already include standard capacity/regularization mechanisms (shrinkage, depth/child-weight limits, row/column subsampling, L2 penalty) and native handling of missing values; no feature scaling was required and no hyperparameter optimization was performed. To limit overfitting, we applied shuffled 10-fold cross-validation on the training set for model selection, evaluated once on the held-out test set, and confirmed performance on the untouched validation set. Model parsimony was enforced via a prespecified stepwise ablation rule (retain additions only if they confer a ≥0.01–0.02 absolute AUROC increase without calibration penalty or materially increasing missingness). For non-tree models, missing data were handled by pairwise complete-case analysis within each evaluated feature set to minimize row loss. Eighty-five candidate clinical and physiologic variables were considered (Table S1).

Model performance was evaluated comprehensively. Discrimination was assessed by the area under the receiver operating characteristic curve (AUC) and the area under the precision–recall curve (AUPRC), while overall accuracy was complemented by the Brier score. Calibration was examined through calibration plots comparing predicted probabilities with observed outcome frequencies; calibration-in-the-large (intercept) and calibration slope were estimated using logistic recalibration, with a perfectly calibrated model expected to follow the 45-degree reference line. Threshold-specific operating characteristics—including sensitivity, specificity, positive and negative predictive values, and the F1 score—were reported at prespecified cut-points (the default 0.50, the Youden index, and specificity-prioritized thresholds of ≥0.90 and ≥0.95). Clinical utility was evaluated using decision curve analysis (DCA), which quantifies net benefit across a range of threshold probabilities and provides a more nuanced assessment of clinical value than accuracy or AUC alone; the model showing the highest net benefit at clinically relevant thresholds was considered superior.

### 2.5. Development of the Overall Scoring System

SHAP were utilized to improve model transparency by offering an interpretable analysis of feature contributions to predictions [20]. SHAP is a model-agnostic method rooted in game theory that allocates importance values to features according to their marginal contributions to the model’s output. This method ensures local accuracy, consistency, and interpretability, thereby enhancing its adaptability across various modeling frameworks. The SHAP methodology guarantees a fair evaluation of each feature’s impact and facilitates effective tracing of the model’s decision-making process.

Feature importance metrics from the XGBoost model, SHAP value rankings, and domain knowledge were integrated to identify key predictors for score development. These selected variables were incorporated into multiple modeling frameworks, including logistic regression, decision tree, random forest, and XGBoost models, to evaluate comparative predictive performance. Manual ablation testing was conducted to assess the impact of adding or removing features on test-set AUC. The final scoring system, referred to as the E3A score (comprising ECG score, APS-III, and age), was chosen by balancing model performance and parsimony. Logistic regression demonstrated the best predictive performance when incorporating the three selected variables (ECG score, APS-III, and age), and was therefore chosen to develop the final scoring model. A nomogram was constructed to facilitate clinical interpretation.

### 2.6. Feature Ablation for Variable Selection

We conducted a prespecified, clinically informed stepwise ablation to evaluate whether adding predictors to the three-variable E3A model (ECG-derived risk score, APS-III, and age) materially improved performance (Table S4). At each step, logistic regression models were refit on the training data and evaluated in the held-out test set using AUC and calibration (Brier score), with threshold-level operating characteristics (sensitivity, specificity, F1 score) reported for transparency. A priori, an added predictor (or bundle of predictors) was retained only if it achieved an absolute AUC gain ≥0.01–0.02 and did not worsen calibration or materially increase missingness/data requirements.

Using this procedure, the baseline E3A achieved AUC 0.806 (Brier 0.096). Adding the Charlson Comorbidity Index yielded a small increment (AUC ~0.816) without threshold-metric gains. The largest observed increase occurred when Charlson, arterial pO_2_, and acute kidney injury (AKI) stage were added together (AUC ~0.827), but calibration deteriorated (Brier ~0.104–0.107) and data completeness decreased. Further sequential additions (e.g., lactate dehydrogenase, chloride, weight, hematocrit, red cell distribution width, albumin, heart failure, red blood cell count, activated partial thromboplastin time, white blood cell count, vasopressor use) produced no durable benefit (AUC plateau/decline, Brier ~0.14). Because APS-III already encodes acute physiologic derangements (including surrogates of oxygenation and renal dysfunction) and correlates with comorbidity burden, added variables contributed limited non-overlapping signal while increasing complexity and casewise deletion (Supplementary Table S4). The proportion of missing values for each study variable, overall and stratified by data subset (train, test, and validation cohorts), is shown in Supplementary Table S5. We did not impute missing data. The finalized E3A score uses three fully observed predictors (ECG-derived risk, APS-III, age), and variable screening relied on XGBoost, which natively handles missingness without distributional assumptions. Predictors with substantial missingness (e.g., LDH, albumin, PaO_2_) offered only minimal incremental discrimination in ablation analyses, so imputation was unlikely to change conclusions while adding complexity and assumption-based bias.

A robustness check excluding age showed that the two-variable model (ECG Score Plus APS-III) performed slightly worse than the three-variable E3A. In the test set, AUROC decreased from 0.806 (E3A) to 0.792, with similar calibration (Brier 0.096 vs. 0.098). These results indicate that age contributes independent signal beyond APS-III and the ECG-derived score. Accordingly, the prespecified parsimony criterion selected the three-variable E3A specification for subsequent validation and reporting.

### 2.7. Data Analysis

All machine learning models were implemented and tested in Python (version 3.12.3). Model performance was assessed through standard metrics including AUC, calibration curves, and other classification indicators as appropriate. Logistic regression analyses, forest plot generation, DCA, and nomogram construction were carried out in R software (version 4.4.2). Baseline variables were described as mean ± standard deviation (SD), median with interquartile range (Q1–Q3), or frequency with percentage (%), depending on variable distribution. Descriptive statistics and summary tables were generated in Python. For the initial XGBoost model used in feature selection, missing values were retained without imputation, leveraging the model’s built-in handling of missing data. However, for the scoring models used to predict 28-day mortality—such as logistic regression, decision tree, and random forest—cases with missing data were excluded from analysis, given these models do not accommodate missing input values. All statistical tests were two-sided. A two-tailed *p*-value below 0.05 was regarded as statistically significant.

## 3. Results

### 3.1. Baseline Characteristics

A total of 18,256 ICU patients were included in the final cohort, stratified by 28-day mortality: 2412 non-survivors (13.2%) and 15,844 survivors (86.8%) (Table 1). Non-survivors were significantly older than survivors, with a median age of 75.0 years (IQR: 63.0–84.0) compared to 67.0 years (IQR: 56.0–78.0). Males accounted for 58.9% of the overall population, with a higher proportion among survivors (59.6%) than non-survivors (54.4%).

Non-survivors exhibited a significantly greater burden of comorbidities (Table S2), including higher rates of heart failure and acute myocardial infarction. In addition, clinical severity scores and ECG-derived risk scores were consistently higher in non-survivors compared to survivors. Median values for key variables were as follows: ECG risk score (24.4 vs. 13.5), Charlson Comorbidity Index (7.0 vs. 5.0), OASIS (38.0 vs. 32.0), SOFA (7.0 vs. 4.0), SAPS-II (47.0 vs. 34.0), and APS-III (59.0 vs. 38.0). All differences were statistically significant (*p* < 0.001).

### 3.2. Calculation of ECG-Based Risk Score

The dataset was divided into training, validation, and test sets in a ratio of 7:1:2. To optimize classification performance, binary cross-entropy was used to calculate the model loss value during training, and the Adam optimizer was employed to update model parameters by minimizing the loss. The key hyperparameters included a batch size of 256, a learning rate of 1 × 10^−4^, and a learning rate decay of 1 × 10^−4^. The model was implemented using PyTorch version 1.13.0 and trained on a server equipped with four NVIDIA RTX 3090 GPUs. After 100 training epochs, the final model achieved an accuracy of 0.813 and an AUC of 0.697 on the test set (Figure S2, Table S3). To benchmark performance, several alternative deep neural network architectures were trained on the same dataset. Among them, the proposed model demonstrated the highest test-set accuracy and sensitivity (Table S3), supporting its selection for ECG-based mortality risk scoring.

### 3.3. Screening Variables Using the XGBoost Model

Using the full set of 85 candidate clinical variables, the XGBoost model achieved strong predictive performance for 28-day mortality, with an AUC of 0.870 (95% CI: 0.853–0.885) in the test set and 0.888 (95% CI: 0.866–0.907) in the validation set (Figure S3). Calibration plots (Figure S4) indicated good agreement between predicted and observed outcomes, with Brier scores of 0.086 and 0.081 in the test and validation sets, respectively.

**Figure 2:**
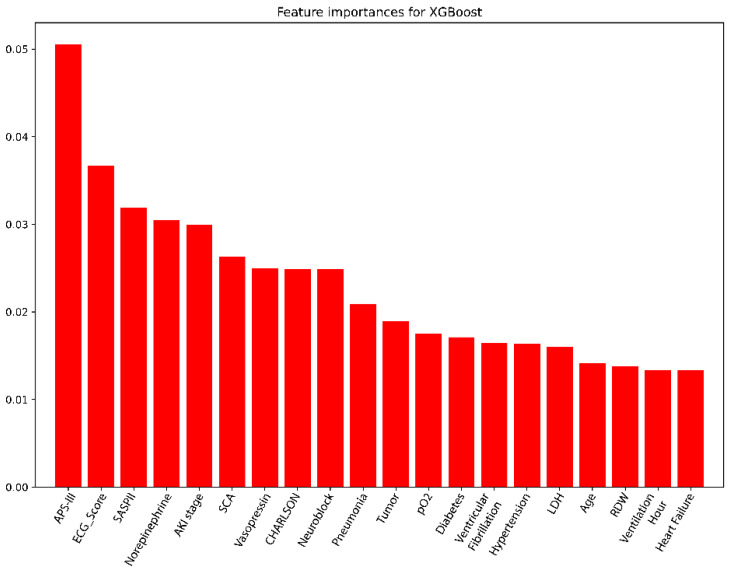
Feature importance values for the top 20 risk factors of the XGBoost model (85 candidate variables) in the training set. Abbreviations: AKI: acute kidney injury. SCA: sudden cardiac arrest. pO2: partial pressure of oxygen in arterial blood. LDH: Lactate dehydrogenase. RDW: Red blood cell distribution width.

Feature importance was evaluated using the trained XGBoost model on the training dataset. As shown in Figure 2, the most influential predictors were the APS-III score and ECG-derived risk score. Other variables with high predictive value included the use of specific vasopressors (e.g., norepinephrine), key comorbidities (e.g., acute kidney injury), laboratory indicators (e.g., lactate dehydrogenase), and demographic features (e.g., age).

**Figure 3:**
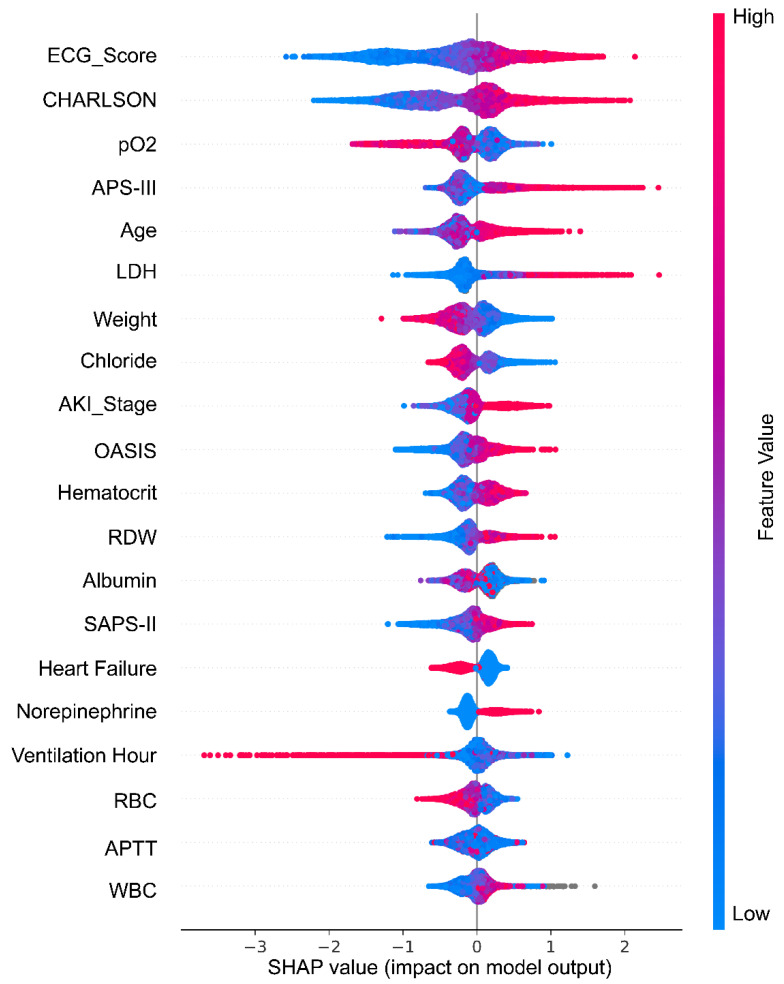
SHAP summary plot for the top 20 predictors of the XGBoost model (85 candidate variables) in the training set. Abbreviations: APTT: activated partial thromboplastin time. LDH: lactate dehydrogenase. pO2: partial pressure of oxygen in arterial blood. RBC: red blood cell. RDW: red blood cell distribution width. WBC: white blood cell.

To further evaluate the contribution of individual features to model predictions, a SHAP summary plot was generated for the top 20 risk factors (Figure 3). This visualization illustrates both the importance and directionality of each feature’s impact on the predicted risk of 28-day mortality. Notably, higher values of ECG score, APS-III and age were strongly associated with increased predicted mortality, reinforcing their critical influence in the model.

These findings highlight the significant contributions of the ECG score, APS-III and age as key predictors of patient outcomes. Their prominence in the analysis underscores their critical role as risk factors and justifies their inclusion in the final predictive model.

### 3.4. Derivation and Evaluation of the 28-Day Mortality Score

The final predictive model, referred to as the E3A Score, was constructed using three variables: ECG score, APS-III, and age. These features were selected based on a combination of SHAP importance rankings and clinical relevance, prioritizing interpretability and integration with existing ICU risk assessment practices. While some variables ranked higher than APS-III and age in SHAP analysis, the selected features offered a favorable balance between predictive power, physiological coverage, and clinical usability.

**Figure 4:**
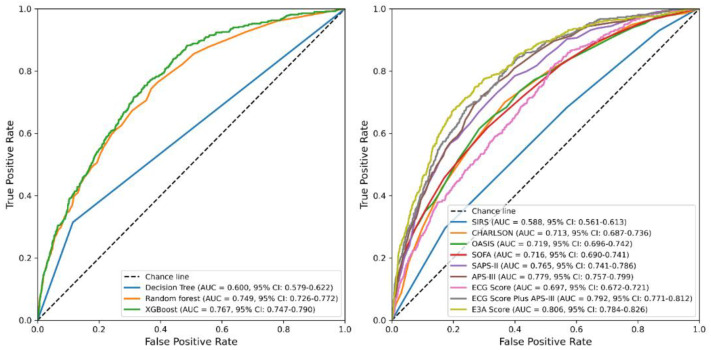
ROC curves for model performance in the test set. **Left panel**: ROC curves comparing the performance of three machine learning models (decision tree, random forest, and XGBoost) based on the three-variable E3A Score. **Right panel**: ROC curves comparing logistic regression models based on traditional clinical scoring systems and ECG-integrated predictors. E3A Score: ECG Score, APS-III, Age.

**Figure 5:**
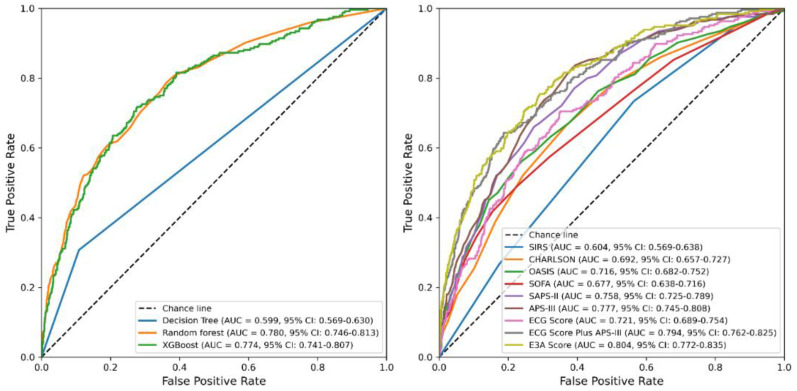
ROC curves for model performance in the validation set. **Left panel**: ROC curves comparing the performance of three machine learning models (decision tree, random forest, and XGBoost) based on the three-variable E3A Score. **Right panel**: ROC curves comparing logistic regression models based on traditional clinical scoring systems and ECG-integrated predictors. E3A Score: ECG Score, APS-III, Age.

**Figure 6:**
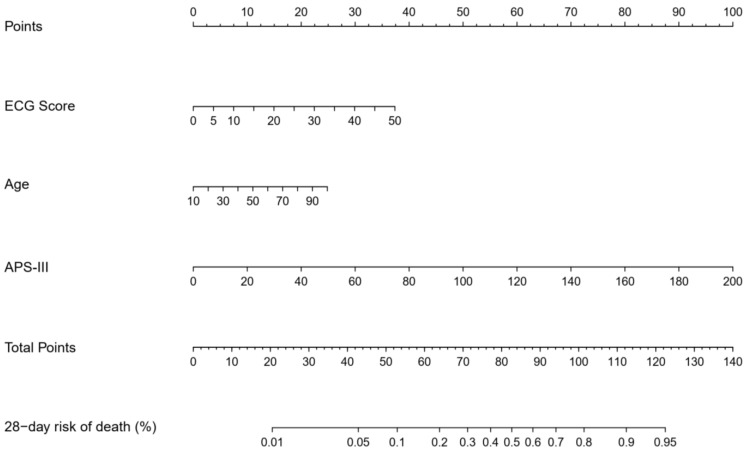
Nomogram of the logistic regression model (E3A Score) for predicting 28-day mortality in ICU patients based on the training set.

Among the four machine learning algorithms evaluated using these three predictors, logistic regression demonstrated the best overall performance, achieving an AUC of 0.806 (95% CI: 0.784–0.826) in the test set (Figure 4, Table 2) and 0.804 (95% CI: 0.772–0.835) in the validation set (Figure 5, Table 3), outperforming decision trees, random forests, and XGBoost. Accordingly, logistic regression was chosen as the final model for deriving the E3A Score, which was visualized as a nomogram to facilitate clinical interpretation (Figure 6). Threshold-dependent operating characteristics for E3A at prespecified cut-points (default 0.50, Youden, Spec ≥ 0.90/0.95) are summarized for both test and validation cohorts in Supplementary Table S6, demonstrating consistent sensitivity–specificity trade-offs and supporting use-case–aligned deployment.

**Figure 7:**
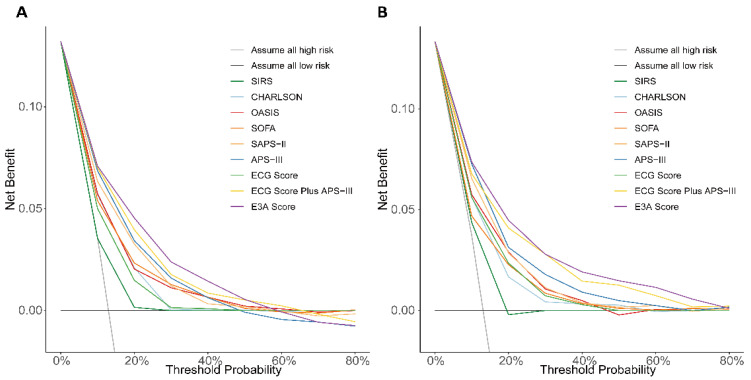
Decision curve analysis of different scoring systems. DCA curves comparing the net clinical benefit of various scoring models for predicting 28-day mortality in the test set (**A**) and validation set (**B**).

DCA demonstrated that the E3A Score provided the highest net clinical benefit across a wide range of threshold probabilities when compared to traditional scoring systems (Figure 7). Calibration plots demonstrated good concordance between predicted and observed 28-day mortality in both the test and validation sets (Figures S5 and S6), with Brier scores of 0.096 and 0.093, respectively, indicating adequate model calibration.

### 3.5. Multivariable Logistic Regression Analysis

Multivariable logistic regression analysis (Figure S7) confirmed the independent predictive value of all three variables included in the E3A scoring system. The ECG score was significantly associated with increased mortality risk, with an odds ratio (OR) of 1.06 per unit increase (95% CI: 1.05–1.06, *p* < 0.001). Age demonstrated a clear dose–response relationship, with an OR of 1.02 per year (95% CI: 1.02–1.02, *p* < 0.001). The APS-III score also remained an independent predictor of 28-day mortality, with an OR of 1.04 per unit increase (95% CI: 1.03–1.04, *p* < 0.001), reinforcing its role as a comprehensive marker of physiological derangement

## 4. Discussion

In this study, we developed a novel machine learning–based model to predict 28-day mortality in ICU patients by integrating an ECG-derived risk score with conventional APS-III score. A total of 85 variables, including the first ECG recorded upon ICU admission, were evaluated and screened. The final model, termed the E3A Score, demonstrated strong predictive performance and outperformed several established ICU severity scoring systems. Notably, the inclusion of the ECG score, selected based on SHAP analysis and clinical relevance, significantly enhanced model discrimination, highlighting the underutilized prognostic value of routinely collected ECG data in critical care settings.

Several conventional severity scoring systems, including APS-III, SAPS-II, SOFA, OASIS, the Charlson Comorbidity Index, GCS and SIRS, have been widely adopted for ICU risk stratification and are supported by extensive validation in clinical research and practice [6,21]. Among these, both APS-III and SAPS-II were designed to capture acute physiological derangement but differ in structure, granularity, and clinical application. SAPS II-incorporates 12 key variables, including age and comorbidities, and offers a simplified, widely applicable model for mortality prediction [5,22]. In contrast, APS-III includes a more comprehensive physiological dataset, such as acid–base balance, oxygenation status, and urine output, allowing for a more nuanced assessment of organ dysfunction severity [23,24]. Other scoring systems, such as SOFA, focus on tracking progressive organ failure and are more suitable for dynamic patient monitoring than baseline risk prediction. The OASIS score emphasizes parsimony and real-time usability by including fewer variables, albeit sometimes at the expense of predictive accuracy. The Charlson Comorbidity Index and SIRS criteria reflect chronic disease burden and inflammatory response, respectively, but are less sensitive to acute physiological status [4].

In our analysis, APS-III and SAPS-II demonstrated moderate discriminative ability for 28-day mortality, with AUCs of 0.779 and 0.765, respectively, in the test set, and 0.777 and 0.758 in the validation set. Other scoring systems showed lower predictive performance. Based on its broader physiological coverage and stronger validation performance, APS-III was ultimately selected for inclusion in the E3A Score.

Importantly, none of the conventional ICU risk scores incorporates direct electrocardiographic data, despite its ability to capture clinically relevant signs of myocardial ischemia, electrical instability, and autonomic dysregulation. ECG is one of the most widely available, low-cost, and noninvasive diagnostic tools in critical care and provides real-time insights into cardiac electrophysiology, ventricular remodeling, and ischemic burden [25]. However, its use in risk scoring models has been historically limited due to challenges in standardized interpretation and computational feature extraction. Accurate ECG analysis typically requires cardiology expertise, which is not consistently available in many ICU environments, particularly in resource-constrained settings.

By leveraging deep learning to quantify ECG-based risk features, our approach automatically extracts prognostically relevant patterns across multiple leads and temporal domains, capturing correlations and transitions in P-waves, QRS complexes, ST-segments, and T-wave morphology [26,27]. The trained model can identify subtle electrocardiographic indicators of mortality risk that may be overlooked by non-cardiologists or clinicians unfamiliar with detailed ECG interpretation [15,28,29,30]. Importantly, the ECG score was derived in an end-to-end manner without predefining specific features, ensuring flexibility in detecting both conventional and less apparent signals. In practice, the convolutional network may capture prognostically relevant patterns such as mean heart rate, rhythm irregularity, QRS widening, bundle branch block, ST-segment elevation or depression, and T-wave abnormalities, while also retaining the ability to identify more subtle or complex waveform changes that are not readily apparent to clinicians. While this design enhances predictive power, it also limits direct attribution of risk to individual ECG components. Future work should therefore incorporate explainability techniques (e.g., saliency mapping, feature attribution, or lead-wise ablation) to clarify which ECG characteristics contribute most strongly to outcome prediction and to further improve the interpretability and clinical acceptance of the model. The ECG-based risk score alone demonstrated strong discriminative ability (AUC = 0.697 in the test set and 0.721 in the validation set) and, when combined with the APS-III score in a multimodal framework, further improved predictive performance (AUC = 0.792 in the test set and AUC = 0.794 in the validation set). This suggests that ECG provides an orthogonal and clinically meaningful dimension of risk stratification, especially when integrated with conventional physiologic severity indices.

To enhance model performance beyond ECG and APS-III, we incorporated the additional variable: age, which was selected based on a combination of SHAP-based importance ranking and supporting clinical evidence. Age is a well-established predictor of poor outcomes in critical illness, reflecting physiological frailty, diminished reserve, and increased susceptibility to organ failure [31,32,33]. The inclusion of the variable improved the AUC and clinical net benefit of the final model, highlighting the strength of combining data-driven selection with domain expertise to achieve both performance and interpretability.

To evaluate the predictive utility of the selected variables, we compared four machine learning algorithms: logistic regression, decision tree, random forest, and XGBoost. While tree-based ensemble methods like random forest and XGBoost are capable of modeling complex non-linear relationships, they may be prone to overfitting in moderately sized datasets and lack transparency in clinical settings. In our cohort, logistic regression outperformed the more complex models in terms of both discrimination (AUC) and calibration, providing the best overall balance between predictive accuracy, interpretability, and clinical applicability. This finding is consistent with a landmark review by Christodoulou et al. [34], which found that machine learning algorithms did not consistently outperform logistic regression across a wide range of clinical prediction models [34,35]. This supports the idea that in structured, low to moderately dimensional healthcare datasets, well-regularized logistic regression remains a competitive and often preferred approach due to its simplicity, transparency, and robustness.

To enhance model transparency, SHAP were employed to provide intuitive, individualized visualizations of how each feature contributed to the predicted risk of 28-day mortality [36,37]. In the SHAP-based feature importance ranking, several variables consistently emerged as key contributors, including the ECG score, APS-III, and age. These variables are consistent with established indicators of illness severity and cardiovascular compromise in critically ill patients. While SHAP analysis offered a quantitative measure of feature contribution, the final variable selection also incorporated clinical judgment, ensuring alignment with physiological plausibility and real-world applicability. This hybrid approach, balancing data-driven insights with domain expertise, allowed the model to achieve both high predictive performance and clinical interpretability [34,38].

E3A’s apparent “low sensitivity” reflects threshold choice rather than an intrinsic limitation and should be aligned to clinical intent. Using our prespecified operating points, early triage can adopt the Youden cut-point (threshold ≈ 0.137), achieving high sensitivity (~0.70–0.75) with moderate specificity (~0.73) and PPV ~0.30, appropriate for low-harm escalation (expedited senior review, repeat lactate/ABG, intensified monitoring) rather than automatic intervention. For rule-out/step-down, a specificity-prioritized cut-point (e.g., Spec ≥ 0.95, threshold ≈ 0.345) yields very high specificity (~0.95) with lower sensitivity (~0.30) and higher PPV (~0.48), supporting conservative de-intensification when otherwise appropriate; a balanced option (Spec ≥ 0.90, threshold ≈ 0.257) provides sensitivity ~0.46 and specificity ~0.90 (PPV ~0.41). These trade-offs were reproduced in the validation set at the same thresholds, indicating stability. In practice, the tool operationalizes care by (i) flagging “high-risk” patients for timely reassessment and (ii) identifying “low-risk” candidates for bed management and step-down; extreme survival outliers likely reflect treatment effects and merit prospective evaluation. Consistent with TRIPOD, sites should re-threshold or recalibrate to local goals and monitor alert volume/override rates [39]; given class imbalance, we report PPV and F1 alongside AUROC to reflect precision in actionability [40]. The score is advisory and intended to augment—not replace—clinical judgment.

E3A can be implemented as a lightweight electronic health record (EHR) module and as a paper/web nomogram. At ICU admission—and on subsequent data refresh—the system automatically retrieves the first digitized 12-lead ECG together with APS-III and age, computes the predicted risk, and records both the probability and an action band in the chart. Where full integration is not yet available, the nomogram permits bedside entry of the three inputs; if the ECG-derived score is temporarily unavailable, a provisional APS-III ± age estimate can be displayed and automatically superseded once the ECG score is posted. Anticipated barriers and corresponding mitigations include: (i) ECG digitization and data latency, addressed by automated HL7/FHIR ingestion and an asynchronous recomputation queue; (ii) vendor and lead-quality heterogeneity, addressed by standardized preprocessing and site-level calibration before go-live; (iii) missingness, addressed by pairwise, feature-set–specific completion rules and explicit “do-not-use when required inputs are absent” flags; (iv) computational constraints, minimized because logistic scoring is negligible in cost and ECG feature extraction can be batched on existing servers; (v) alert burden, managed by non-interruptive displays with monitoring of alert volume and override rates; (vi) model governance and drift, ensured through versioned outputs, regular calibration audits, and local re-thresholding/recalibration; and (vii) equity, supported by routine performance monitoring across age, sex, race/ethnicity, and admission type. This pathway enables deployment within standard escalation/step-down and bed-management workflows without duplicating existing processes.

Several limitations merit consideration. As a single-database, retrospective analysis based on MIMIC-IV, the study is susceptible to selection and information biases and to temporal and institutional variation; consequently, transportability to ICUs with different case mix, care pathways, and documentation standards remains uncertain. Inputs were limited to the first ICU time window and the first post-admission ECG, yielding a static baseline model that does not capture within-stay dynamics, evolving physiology, or responses to therapy. Although XGBoost tolerated missingness during variable screening, the final parsimonious score used complete-case analysis, which can preferentially exclude sicker patients and distort calibration; reporting variable-level missingness and conducting multiple-imputation sensitivity analyses would strengthen inference. Model performance is threshold-dependent: the default operating point emphasizes specificity, whereas alternative thresholds can raise sensitivity at an expected cost in false positives, implying that site-specific re-thresholding or recalibration will likely be required before clinical use. Heterogeneity in ECG acquisition and preprocessing (vendor, sampling rate, filtering, lead quality) may alter the learned ECG risk representation and warrants explicit robustness checks. Potential performance differentials across demographic and clinical subgroups were not assessed and should be quantified and, where necessary, mitigated through appropriate governance.

To clarify clinical usefulness and generalizability, external, multicenter validation across geographically and operationally diverse ICUs is warranted, with discrimination, calibration-in-the-large and slope, decision-analytic net benefit, and threshold-specific confusion matrices reported; prespecified subgroup and temporal analyses would further assess fairness and stability, and site-level recalibration should be considered where miscalibration is observed. Prospective evaluation could proceed in stages—initial silent electronic health record (EHR) deployment to monitor drift, alert volume, workload, and adjudication without altering care, followed by a pragmatic trial (e.g., a stepped-wedge cluster design) comparing usual care with use of the score at prespecified thresholds aligned with intended use (recall-prioritized for early triage vs. high-specificity for rule-out/step-down). Key outcomes should include 28-day mortality, ICU length of stay, unplanned ICU transfer/readmission, time to escalation, and human-factors and implementation metrics (alert burden, override rates, usability, workflow fit), with health-economic evaluation where feasible. Implementation will also depend on availability of digitized 12-lead ECGs, data latency, computing resources, and integration with clinical systems. The score is intended to augment, not replace, clinical judgment and should not be applied when required inputs are unavailable or outside the defined window. Taken together, these steps are necessary to establish generalizability, safety, and real-world impact.

## 5. Conclusions

In this study, we developed a robust and interpretable machine learning model for predicting 28-day mortality in ICU patients by integrating an ECG-derived risk score with key clinical variables. The resulting E3A Score demonstrated superior discriminatory performance compared to conventional severity scoring systems and highlighted the untapped prognostic value of ECG data. This model may facilitate timely interventions, optimize resource allocation, and improve individualized care for critically ill patients.

## Figures and Tables

**Table 1:** Baseline Characteristics of ICU Patients in the MIMIC-IV Cohort.

	Overall (*n* = 18,256)	Non-Survivors (*n* = 2412)	Survivors (*n* = 15,844)	*p* Value
Age, median [Q1, Q3]	68.0 [57.0, 79.0]	75.0 [63.0, 84.0]	67.0 [56.0, 78.0]	<0.001
Gender, *n* (%)				<0.001
Male	10753 (58.9)	1313 (54.4)	9440 (59.6)	
Female	7503 (41.1)	1099 (45.6)	6404 (40.4)	
ECG Score, median [Q1, Q3]	14.7 [7.9, 24.2]	24.4 [15.6, 33.4]	13.5 [7.2, 22.1]	<0.001
SIRS, median [Q1, Q3]	3.0 [2.0, 3.0]	3.0 [2.0, 4.0]	3.0 [2.0, 3.0]	<0.001
CHARLSON, median [Q1, Q3]	5.0 [3.0, 7.0]	7.0 [5.0, 9.0]	5.0 [3.0, 7.0]	<0.001
OASIS, median [Q1, Q3]	32.0 [26.0, 37.0]	38.0 [32.0, 44.0]	31.0 [25.0, 36.0]	<0.001
SOFA, median [Q1, Q3]	4.0 [2.0, 7.0]	7.0 [4.0, 10.0]	4.0 [2.0, 6.0]	<0.001
SAPS-II, median [Q1, Q3]	36.0 [28.0, 45.0]	47.0 [38.0, 58.0]	34.0 [27.0, 42.0]	<0.001
APS-III, median [Q1, Q3]	41.0 [30.0, 55.0]	59.0 [45.0, 79.0]	38.0 [29.0, 51.0]	<0.001

Note: Patients were stratified by 28-Day Mortality. Values are presented as median [Q1, Q3] for continuous variables and *n* (%) for categorical variables. *p* values reflect comparisons between non-survivors and survivors using the Mann–Whitney U test for continuous variables and the chi-square test for categorical variables. No missing values were present in the listed variables.

**Table 2:** Comparison of predictive performance metrics for machine learning models and logistic regression–based clinical scoring systems in the test set.

Item	Accuracy	PPV	Sensitivity	Specificity	AUC	AUPRC	F1 Score	Brier Score
Machine learning models								
(ECG Score + APS-III + Age)								
Decision Tree	0.81	0.295	0.315	0.885	0.6	0.35	0.304	0.190
Random forest	0.862	0.452	0.197	0.964	0.749	0.333	0.274	0.107
XGBoost	0.867	0.497	0.159	0.975	0.767	0.342	0.241	0.104
Logistic regression models								
SIRS	0.868		0	1	0.588	0.295	0	0.113
CHARLSON	0.868	0.533	0.017	0.998	0.713	0.265	0.032	0.107
OASIS	0.87	0.577	0.062	0.993	0.719	0.311	0.112	0.104
SOFA	0.869	0.530	0.072	0.99	0.716	0.308	0.128	0.105
SAPS-II	0.87	0.534	0.114	0.985	0.765	0.343	0.188	0.102
APS-III	0.867	0.484	0.095	0.985	0.779	0.344	0.159	0.102
ECG Score	0.868		0	1	0.697	0.258	0	0.109
ECG Score Plus APS-III	0.873	0.576	0.149	0.983	0.792	0.377	0.237	0.098
E3A Score	0.873	0.578	0.153	0.983	0.806	0.399	0.242	0.096

E3A Score refers to the combined model of ECG Score, APS-III, and age. ECG Score Plus APS-III refers to the two-variable combined model comprising the ECG-derived risk score and APS-III, with age excluded. PPV, positive predictive value; AUC, area under the receiver operating characteristic curve; AUPRC, area under the precision–recall curve.

**Table 3:** Comparison of predictive performance metrics for machine learning models and logistic regression–based clinical scoring systems in the validation set.

Item	Accuracy	PPV	Sensitivity	Specificity	AUC	AUPRC	F1 Score	Brier Score
Machine learning models								
(ECG Score + APS-III + Age)								
Decision Tree	0.813	0.304	0.307	0.891	0.599	0.352	0.305	0.187
Random forest	0.871	0.543	0.234	0.970	0.780	0.402	0.327	0.099
XGBoost	0.865	0.490	0.197	0.968	0.774	0.374	0.281	0.102
Logistic regression models								
SIRS	0.866		0.000	1.000	0.604	0.283	0.000	0.114
CHARLSON	0.869	0.727	0.033	0.998	0.692	0.273	0.063	0.109
OASIS	0.864	0.389	0.029	0.993	0.716	0.316	0.053	0.105
SOFA	0.867	0.536	0.061	0.992	0.677	0.289	0.110	0.108
SAPS-II	0.868	0.548	0.070	0.991	0.758	0.336	0.124	0.103
APS-III	0.871	0.588	0.123	0.987	0.777	0.373	0.203	0.101
ECG Score	0.866		0.000	1.000	0.721	0.299	0.000	0.107
ECG Score Plus APS-III	0.879	0.667	0.189	0.985	0.794	0.440	0.294	0.095
E3A Score	0.881	0.696	0.197	0.987	0.804	0.466	0.307	0.093

E3A Score refers to the combined model of ECG Score, APS-III, and age. ECG Score Plus APS-III refers to the two-variable combined model comprising the ECG-derived risk score and APS-III, with age excluded. PPV, positive predictive value; AUC, area under the receiver operating characteristic curve; AUPRC, area under the precision–recall curve.

## Data Availability

The data used in this study are available from the publicly accessible MIMIC-IV database (https://physionet.org/content/mimiciv/2.2/, accessed on 16 March 2025). Data used and analyzed during the study are available from the corresponding author upon reasonable request.

## Supplementary Materials

The following supporting information can be downloaded at: https://www.mdpi.com/article/10.3390/jcm14207163/s1, Figure S1: Flowchart of patient selection from the MIMIC-IV database; Figure S2: Model convergence during training; Figure S3: ROC curves of the XGBoost model using 85 candidate variables; Figure S4: Calibration plots of the XGBoost model (85 candidate variables); Figure S5: Calibration plots for predictive models in the test set; Figure S6: Calibration plots for predictive models in the validation set; Figure S7: Multivariable logistic regression assessing the association of ECG Score, APS-III, and Age with 28-day mortality. Table S1: Full List of 85 Candidate Variables Included in the Study; Table S2: Baseline characteristics stratified by 28-day mortality in the MIMIC-IV cohort; Table S3: Performance metrics of different deep neural network models in the test set [41,42,43]; Table S4. Test-set performance of the E3A score and incremental model extensions; Table S5. Missingness of Study Variables Overall and by Data Subset (Train, Test, and Validation Cohorts); Table S6. Threshold selection for the E3A score—test and validation performance.

## Abbreviations

The following abbreviations are used in this manuscript:

APS-III	Acute Physiology Score III
AUC	Area Under Curve
DCA	Decision Curve Analysis
ECG	Electrocardiogram
GCS	Glasgow coma scale
ICU	Intensive Care Unit
ML	Machine Learning
OASIS	Oxford Acute Severity of Illness Score
SAPS-II	Simplified Acute Physiology Score II
SHAP	Shapley Additive exPlanations
SIRS	Systemic Inflammatory Response Syndrome
SOFA	Sequential Organ Failure Assessment
