# Supplementary material for: Interpretable Machine Learning Model Integrating Electrocardiographic and Acute Physiology Metrics for Mortality Prediction in Critical Ill Patients
Source: J Clin Med. 2025 Oct 11;14(20):7163. doi: 10.3390/jcm14207163 (PMC12565335; doi:10.3390/jcm14207163)
Supplement: Supplementary file 1 [file jcm-14-07163-s001.zip › jcm-3853021-supplementary.pdf]

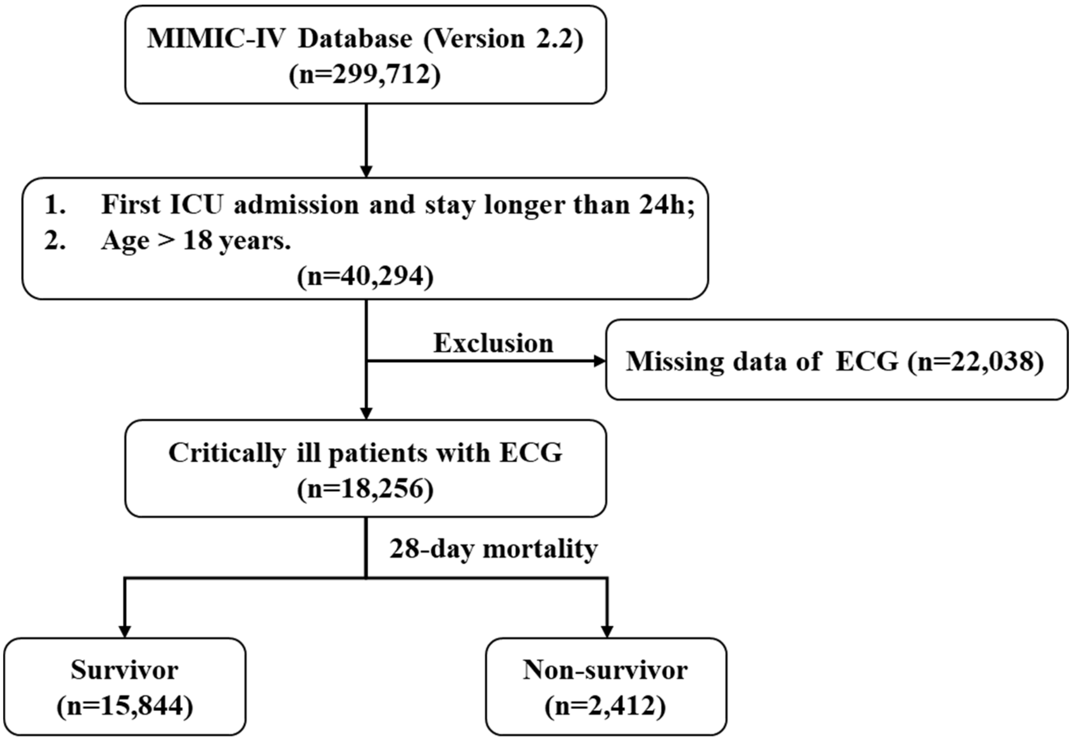


**Supplementary Figure 1.** Flowchart of patient selection from the MIMIC-IV database

**Abbreviations:** ECG: Electrocardiogram. ICU: Intensive care unit.

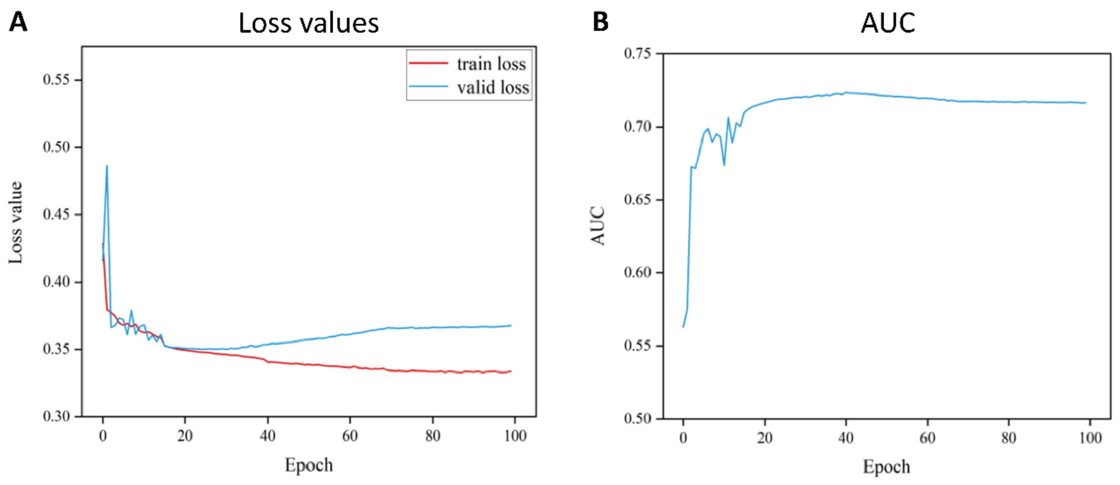


**Supplementary Figure 2.** Model convergence during training

(A) Loss curves for training and validation sets over 100 epochs. (B) Area under the curve (AUC) progression on the validation set during training, demonstrating performance stabilization.

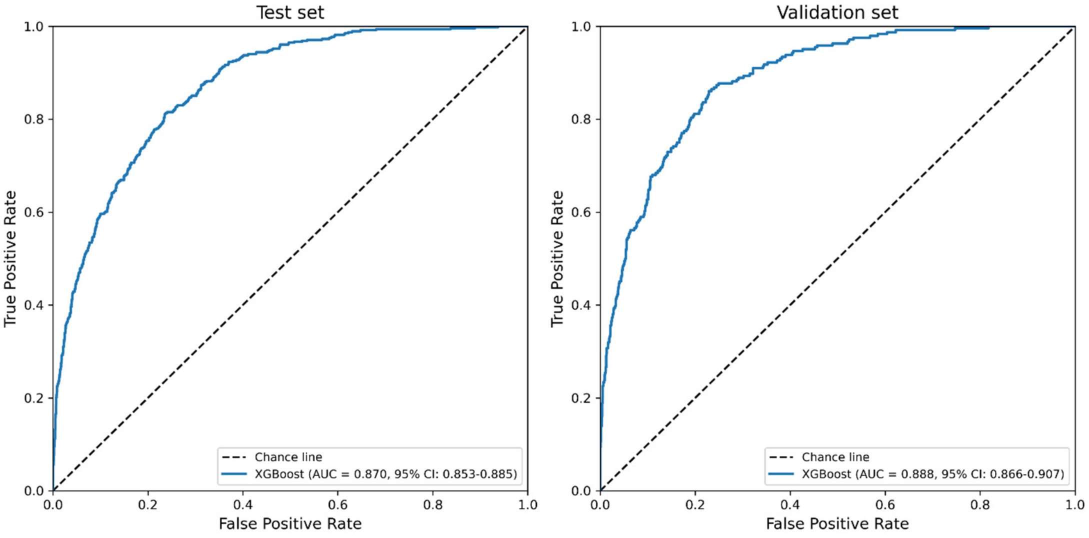


**Supplementary Figure 3.** ROC curves of the XGBoost model using 85 candidate variables

Left panel: Performance in the test set (AUC = 0.870, 95% CI: 0.853–0.885). Right panel: Performance in the validation set (AUC = 0.888, 95% CI: 0.866–0.907).

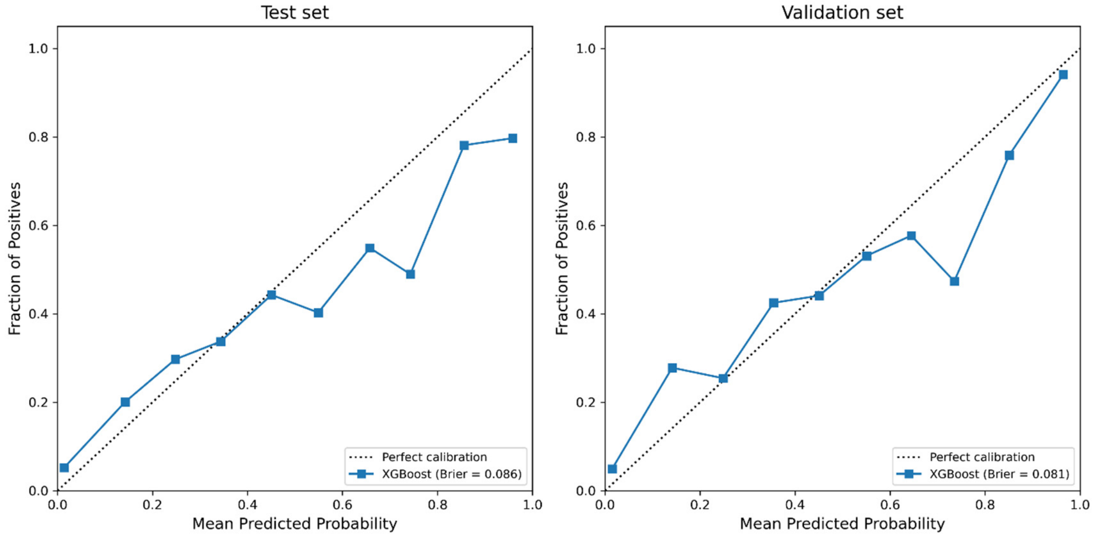


**Supplementary Figure 4.** Calibration plots of the XGBoost model (85 candidate variables)

Left panel: Performance in the test set (Brier score = 0.086). Right panel: Performance in the validation set (Brier score = 0.081).

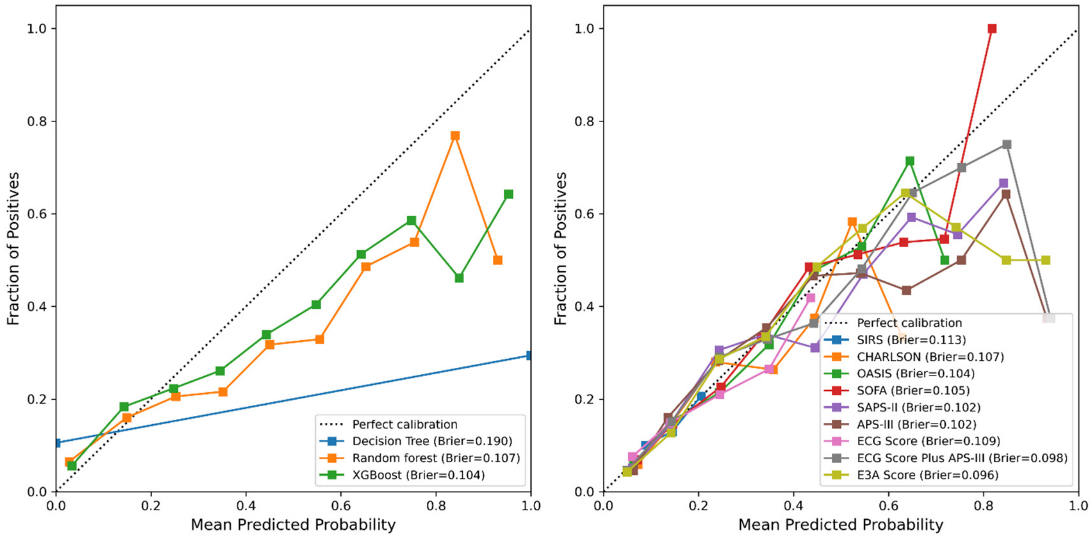


**Supplementary Figure 5.** Calibration plots for predictive models in the test set

Left panel: Calibration curves comparing the performance of three machine learning models (decision tree, random forest, and XGBoost) based on the three-variable E3A Score.

Right panel: Calibration curves comparing logistic regression models based on traditional clinical scoring systems and ECG-integrated predictors.

E3A Score: ECG Score, APS-III, Age.

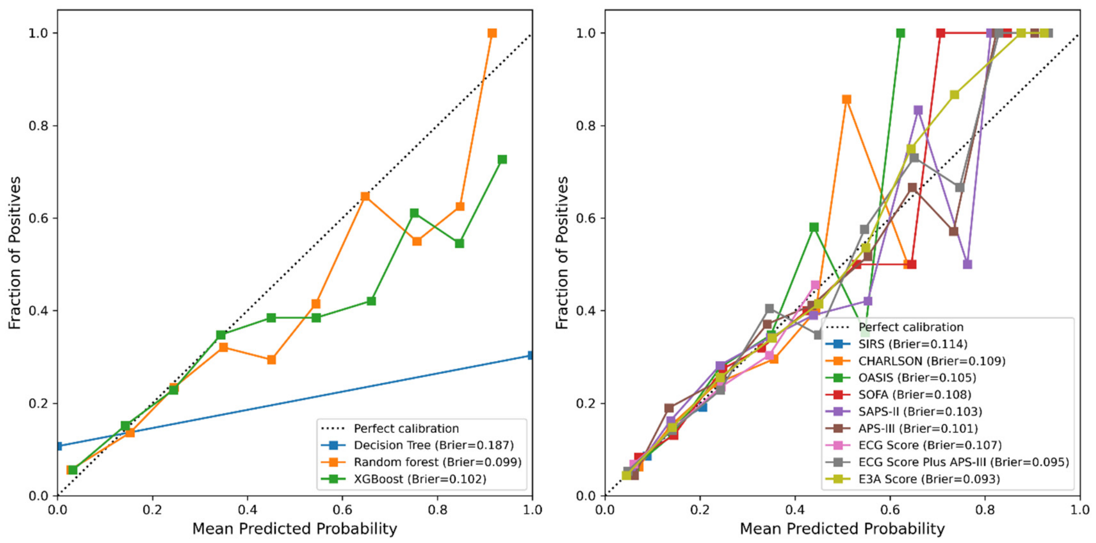


**Supplementary Figure 6.** Calibration plots for predictive models in the validation set

Left panel: Calibration curves comparing the performance of three machine learning models (decision tree, random forest, and XGBoost) based on the three-variable E3A Score.

Right panel: Calibration curves comparing logistic regression models based on traditional clinical scoring systems and ECG-integrated predictors.

E3A Score: ECG Score, APS-III, Age.

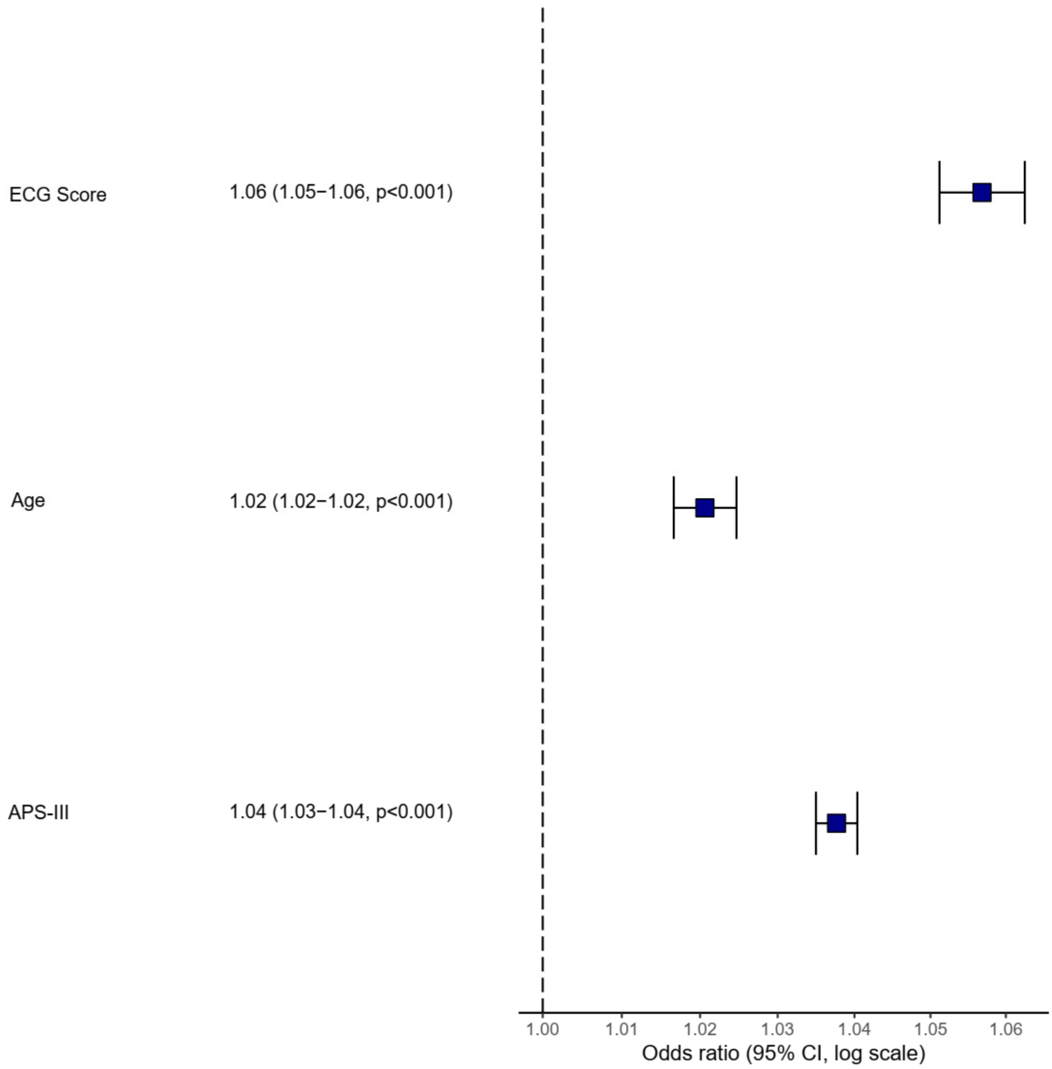


**Supplementary Figure 7.** Multivariable logistic regression assessing the association of ECG Score, APS-III, and Age with 28-day mortality

**Supplementary Table 1.** Full List of 85 Candidate Variables Included in the Study

| Categories | Variables |
|---|---|
| **Demographics** | Ag, Gender, Race, Weight, Height, Body mass index |
| **Vital Signs** | Heart Rate, NSBP, NDBP, NMBP, ASBP, ADBP, AMBP, Respiratory rate, SpO2, Body temperature |
| **ECG and Laboratory Test Results** | ECG Score, White blood cell, Red blood cell, Platelet, Hemoglobin, RDW, Hematocrit, Alanine Aminotransferase, Aspartate Aminotransferase, Albumin, Total bilirubin, Blood urea nitrogen, Creatinine, Lactate dehydrogenase, Creatine Kinase, Creatine Kinase-MB, Troponin T, Sodium, Potassium, Calcium, Chloride, Plasma glucose, Anion gap, pH, pO2, pCO2, tCO2, Lactate in arterial blood, Free calcium in arterial blood, Prothrombin time, Activated partial thromboplastin time, International Normalized Ratio, Fibrinogen |
| **Vasopressors** | Dopamine, Epinephrine, Norepinephrine, Phenylephrine, Neuroblock, Dobutamine, Milrinone, Vasopressin |
| **Severity Scores** | SOFA, APS-III, SIRS, SAPS-II, OASIS, CHARLSON, GCS |
| **Comorbidities and Interventions** | Hypertension, Hyperlipidemia, Diabetes, Heart failure, Tumor, Chronic kidney disease, Cirrhosis, Pneumonia, Acute myocardial infarction, Stroke, Sudden cardiac arrest, Cardiogenic shock, Ventricular tachycardia, Ventricular fibrillation, Sepsis, Acute kidney injury, Acute kidney injury stage, Ventilation, Ventilation time (hour), CRRT, CRRT time (day) |

**Abbreviations:** NSBP: non-invasive systolic blood pressure; NDBP: non-invasive diastolic blood pressure; NMBP: non-invasive mean blood pressure; ASBP: atrial systolic blood pressure; ADBP: atrial diastolic blood pressure; AMBP: atrial mean blood pressure; ECG: electrocardiogram; SpO2: pulse oxygen saturation; RDW: red blood cell distribution width; pO2: partial pressure of oxygen in arterial blood; pCO2: partial pressure of carbon dioxide in arterial blood; tCO2: Total carbon dioxide in arterial blood; CRRT: continuous renal replacement therapy.

**Supplementary Table 2.** Baseline characteristics stratified by 28-day mortality in the MIMIC-IV cohort

| | Overall (n=18256) | Non-survivors (n=2412) | Survivors (n=15844) | P Value |
|---|---|---|---|---|
| BMI (kg/m^2) | 28.16 [24.52, 32.81] | 26.76 [23.06, 31.80] | 28.36 [24.80, 32.90] | <0.001 |
| ASBP (mmHg) | 117.00 [103.00, 135.00] | 115.00 [97.00, 139.00] | 118.00 [104.00, 135.00] | <0.001 |
| ADBP (mmHg) | 60.00 [52.00, 69.00] | 58.00 [49.00, 69.00] | 60.00 [52.00, 69.00] | <0.001 |
| WBC (k/μL) | 10.90 [7.90, 14.90] | 12.40 [8.60, 17.70] | 10.70 [7.90, 14.60] | <0.001 |
| RDW (%) | 14.20 [13.30, 15.60] | 15.30 [14.00, 17.20] | 14.10 [13.20, 15.30] | <0.001 |
| pO2 (mmHg) | 135.00 [71.00, 290.00] | 85.00 [52.00, 152.00] | 154.00 [76.00, 309.00] | <0.001 |
| Lactate (mmol/L) | 1.80 [1.20, 2.60] | 2.10 [1.40, 3.40] | 1.80 [1.20, 2.50] | <0.001 |
| BUN (mg/dL) | 19.00 [13.00, 30.00] | 29.00 [18.00, 49.00] | 18.00 [13.00, 28.00] | <0.001 |
| Creatinine (mg/dL) | 1.00 [0.70, 1.40] | 1.30 [0.80, 2.10] | 0.90 [0.70, 1.30] | <0.001 |
| Hypertension | 8347 (45.72) | 954 (39.55) | 7393 (46.66) | <0.001 |
| Hyperlipidemia | 7182 (39.34) | 775 (32.13) | 6407 (40.44) | <0.001 |
| Diabetes | 32.0 [26.0,37.0] | 38.0 [32.0,44.0] | 31.0 [25.0,36.0] | 0.278 |
| Heart Failure | 5336 (29.23) | 886 (36.73) | 4450 (28.09) | <0.001 |
| Tumor | 2920 (15.99) | 572 (23.71) | 2348 (14.82) | <0.001 |
| AMI | 2716 (14.88) | 427 (17.70) | 2289 (14.45) | <0.001 |

Continuous variables are presented as median [Q1, Q3], and categorical variables are expressed as number (percentage).

**Abbreviation:** AMI: Acute myocardial infarction. ASBP: Atrial systolic blood pressure. ADP (mmHg): Atrial diastolic blood pressure. BMI: Body mass index. RDW: Red blood cell distribution width. WBC: White blood cell.

**Supplementary Table 3.** Performance metrics of different deep neural network models in the test set

| Networks | Accuracy | Sensitivity | Specificity | F1 score | AUC |
|---|---|---|---|---|---|
| ResNet1d_wang [1] | 0.768 | 0.537 | 0.774 | 0.613 | 0.659 |
| Inception1d [2] | 0.739 | 0.534 | 0.834 | 0.618 | 0.683 |
| XresNet101 [3] | 0.769 | 0.538 | 0.775 | 0.615 | 0.663 |
| Proposed Model | 0.813 | 0.555 | 0.735 | 0.623 | 0.697 |

**Supplementary Table 4. Test-set performance of the E3A score and incremental model extensions.**

| No. | Model specification (incremental additions to E3A) | Accuracy | PPV | Sensitivity | Specificity | AUC | AUPRC | F1 Score | Brier Score |
|---|---|---|---|---|---|---|---|---|---|
| 0 | ECG Score Plus APS-III (ECG-derived risk score + APS-III) | 0.873 | 0.576 | 0.149 | 0.983 | 0.792 | 0.377 | 0.237 | 0.098 |
| 1 | E3A (ECG-derived risk score + APS-III + age) | 0.873 | 0.578 | 0.153 | 0.983 | 0.806 | 0.399 | 0.242 | 0.096 |
| 2 | E3A + Charlson Comorbidity Index | 0.876 | 0.613 | 0.174 | 0.983 | 0.816 | 0.417 | 0.271 | 0.094 |
| 3 | E3A + Charlson Comorbidity Index + arterial $pO_2$ | 0.856 | 0.632 | 0.202 | 0.978 | 0.816 | 0.455 | 0.307 | 0.107 |
| 4 | E3A + Charlson Comorbidity Index + arterial $pO_2$ + acute kidney injury (AKI) stage | 0.857 | 0.630 | 0.216 | 0.976 | 0.827 | 0.477 | 0.322 | 0.104 |
| 5 | E3A + Charlson Comorbidity Index + arterial $pO_2$ + AKI stage + lactate dehydrogenase (LDH) | 0.791 | 0.585 | 0.232 | 0.953 | 0.787 | 0.505 | 0.332 | 0.142 |
| 6 | E3A + Charlson Comorbidity Index + arterial $pO_2$ + AKI stage + LDH + chloride | 0.791 | 0.585 | 0.232 | 0.953 | 0.787 | 0.505 | 0.332 | 0.142 |
| 7 | E3A + Charlson Comorbidity Index + arterial $pO_2$ + AKI stage + LDH + chloride + weight | 0.791 | 0.585 | 0.232 | 0.953 | 0.787 | 0.505 | 0.332 | 0.142 |
| 8 | E3A + Charlson Comorbidity Index + arterial $pO_2$ + AKI stage + LDH + chloride + weight + hematocrit (Hct) | 0.790 | 0.579 | 0.226 | 0.953 | 0.786 | 0.503 | 0.325 | 0.142 |
| 9 | E3A + Charlson Comorbidity Index + arterial $pO_2$ + AKI stage + LDH + chloride + weight + Hct + red cell distribution width (RDW) | 0.793 | 0.591 | 0.242 | 0.952 | 0.789 | 0.509 | 0.343 | 0.141 |

| | | | | | | | | | |
|---|---|---|---|---|---|---|---|---|---|
| 10 | E3A + Charlson Comorbidity Index + arterial $pO_2$ + AKI stage + LDH + chloride + weight + Hct + RDW + albumin | 0.793 | 0.591 | 0.242 | 0.952 | 0.789 | 0.509 | 0.343 | 0.141 |
| 11 | E3A + Charlson Comorbidity Index + arterial $pO_2$ + AKI stage + LDH + chloride + weight + Hct + RDW + albumin + heart failure (HF) | 0.797 | 0.607 | 0.265 | 0.951 | 0.792 | 0.510 | 0.369 | 0.141 |
| 12 | E3A + Charlson Comorbidity Index + arterial $pO_2$ + AKI stage + LDH + chloride + weight + Hct + RDW + albumin + HF + red blood cell (RBC) count | 0.795 | 0.594 | 0.265 | 0.948 | 0.790 | 0.508 | 0.366 | 0.141 |
| 13 | E3A + Charlson Comorbidity Index + arterial $pO_2$ + AKI stage + LDH + chloride + weight + Hct + RDW + albumin + HF + RBC count + activated partial thromboplastin time (APTT) | 0.797 | 0.612 | 0.271 | 0.950 | 0.788 | 0.511 | 0.375 | 0.141 |
| 14 | E3A + Charlson Comorbidity Index + arterial $pO_2$ + AKI stage + LDH + chloride + weight + Hct + RDW + albumin + HF + RBC count + APTT + white blood cell (WBC) count | 0.797 | 0.606 | 0.284 | 0.946 | 0.788 | 0.508 | 0.387 | 0.141 |
| 15 | E3A + Charlson Comorbidity Index + arterial $pO_2$ + AKI stage + LDH + chloride + weight + Hct + RDW + albumin + HF + RBC count + APTT + WBC count + norepinephrine use | 0.797 | 0.595 | 0.310 | 0.939 | 0.791 | 0.513 | 0.408 | 0.141 |
| 16 | E3A + Charlson Comorbidity Index + arterial $pO_2$ + AKI stage + LDH + chloride + weight + Hct + RDW + albumin + HF + RBC count + APTT + WBC count + norepinephrine use + vasopressin use | 0.801 | 0.614 | 0.310 | 0.943 | 0.794 | 0.522 | 0.412 | 0.140 |

E3A Score refers to the combined model of ECG Score, APS-III, and age.

ECG Score Plus APS-III refers to the two-variable combined model comprising the ECG-derived risk score and APS-III, with age excluded.

PPV, positive predictive value; AUC, area under the receiver operating characteristic curve; AUPRC, area under the precision–recall curve.

**Supplementary Table 5. Missingness of Study Variables Overall and by Data Subset (Train, Test, and Validation Cohorts).**

| Variable | Overall Missing n(%) | Train Missing n(%) | Test Missing n(%) | Validation Missing n(%) |
|---|---|---|---|---|
| Lactate Dehydrogenase | 9532 (52.2) | 6655 (52.1) | 1922 (52.6) | 955 (52.3) |
| Serum Albumin | 9354 (51.2) | 6557 (51.3) | 1856 (50.8) | 941 (51.5) |
| Arterial partial pressure of oxygen ($PaO_2$) | 4968 (27.2) | 3520 (27.5) | 950 (26.0) | 498 (27.3) |
| Activated Partial Thromboplastin Time | 1207 (6.6) | 865 (6.8) | 233 (6.4) | 109 (6.0) |
| Body Weight | 124 (0.7) | 89 (0.7) | 23 (0.6) | 12 (0.7) |
| White Blood Cell Count | 90 (0.5) | 57 (0.4) | 22 (0.6) | 11 (0.6) |
| Red Cell Distribution Width | 90 (0.5) | 59 (0.5) | 21 (0.6) | 10 (0.5) |
| Hematocrit | 100 (0.5) | 64 (0.5) | 25 (0.7) | 11 (0.6) |
| Red Blood Cell Count | 82 (0.4) | 53 (0.4) | 20 (0.5) | 9 (0.5) |
| Serum Chloride | 42 (0.2) | 27 (0.2) | 13 (0.4) | 2 (0.1) |
| Norepinephrine use (Yes/No) | 0 (0.0) | 0 (0.0) | 0 (0.0) | 0 (0.0) |
| Heart Failure (Yes/No) | 0 (0.0) | 0 (0.0) | 0 (0.0) | 0 (0.0) |
| Age | 0 (0.0) | 0 (0.0) | 0 (0.0) | 0 (0.0) |
| Acute Physiology Score III (APS-III) | 0 (0.0) | 0 (0.0) | 0 (0.0) | 0 (0.0) |
| Simplified Acute Physiology Score II (SAPS-II) | 0 (0.0) | 0 (0.0) | 0 (0.0) | 0 (0.0) |
| Sequential Organ Failure Assessment (SOFA) | 0 (0.0) | 0 (0.0) | 0 (0.0) | 0 (0.0) |
| Oxford Acute Severity of Illness Score (OASIS) | 0 (0.0) | 0 (0.0) | 0 (0.0) | 0 (0.0) |
| Charlson Comorbidity Index | 0 (0.0) | 0 (0.0) | 0 (0.0) | 0 (0.0) |

| | | | | |
|---|---|---|---|---|
| Systemic Inflammatory Response Syndrome (SIRS) | 0 (0.0) | 0 (0.0) | 0 (0.0) | 0 (0.0) |
| Acute Kidney Injury Stage | 0 (0.0) | 0 (0.0) | 0 (0.0) | 0 (0.0) |
| Sex (Male / Female) | 0 (0.0) | 0 (0.0) | 0 (0.0) | 0 (0.0) |
| Vasopressin use (Yes/No) | 0 (0.0) | 0 (0.0) | 0 (0.0) | 0 (0.0) |

**Supplementary Table 6. Threshold selection for the E3A score—test and validation performance.**

| Threshold criterion | Threshold | Sensitivity | Specificity | PPV | F1 Score |
|---|---|---|---|---|---|
| **Test set** | | | | | |
| Default 0.50 | 0.5 | 0.153 | 0.983 | 0.578 | 0.242 |
| Youden's Index (max) | 0.137 | 0.747 | 0.731 | 0.297 | 0.425 |
| Specificity ≥ 0.90 | 0.257 | 0.455 | 0.902 | 0.414 | 0.433 |
| Specificity ≥ 0.95 | 0.345 | 0.304 | 0.95 | 0.484 | 0.374 |
| **Validation set** | | | | | |
| Default 0.50 | 0.5 | 0.197 | 0.987 | 0.696 | 0.307 |
| Youden's Index (max) | 0.137 | 0.709 | 0.748 | 0.302 | 0.424 |
| Specificity ≥ 0.90 | 0.257 | 0.459 | 0.91 | 0.439 | 0.449 |
| Specificity ≥ 0.95 | 0.345 | 0.352 | 0.951 | 0.524 | 0.422 |

E3A Score refers to the combined model of ECG Score, APS-III, and age. PPV, positive predictive value.

**References:**

1. Wang Z, Yan W, Oates T. Time series classification from scratch with deep neural networks: A strong baseline. In: 2017 International Joint Conference on Neural Networks (IJCNN). Anchorage, AK, USA: IEEE; 2017. p. 1578–85.

2. Ismail Fawaz H, Lucas B, Forestier G, Pelletier C, Schmidt DF, Weber J, et al. InceptionTime: Finding AlexNet for time series classification. Data Min Knowl Disc. 2020;34:1936–62.

3. Strodthoff N, Wagner P, Schaeffter T, Samek W. Deep Learning for ECG Analysis: Benchmarks and Insights from PTB-XL. IEEE J Biomed Health Inform. 2021;25:1519–28.
